# Bidirectional Ventricular Tachycardia in Acute Multivessel Myocardial Infarction

**DOI:** 10.7759/cureus.25845

**Published:** 2022-06-11

**Authors:** Frank Hsu, Justin Goh, Andrew Jung, Milan Patel, Rami Akel, Kenneth Yamamura

**Affiliations:** 1 Internal Medicine, Hospital Corporation of America (HCA) Healthcare/University of South Florida (USF) Morsani College of Medicine Graduate Medical Education (GME) Programs at Regional Medical Center Bayonet Point, Hudson, USA; 2 Cardiology, Hospital Corporation of America (HCA) Healthcare/University of South Florida (USF) Morsani College of Medicine Graduate Medical Education (GME) Programs at Regional Medical Center Bayonet Point, Hudson, USA; 3 Cardiology, Graduate Medical Education (GME), Hospital Corporation of America (HCA) Healthcare/University of South Florida (USF) Morsani College of Medicine Graduate Medical Education (GME) Programs at Regional Medical Center Bayonet Point, Hudson, USA

**Keywords:** alternating left and right bundle branch blocks, ventricular dysrhythmia, triggered activity, reentry, type i myocardial infarction, automaticity

## Abstract

Bidirectional ventricular tachycardia (BVT) is a rare and unusual ventricular dysrhythmia that is characterized by a beat-to-beat alternation of the QRS axis. This can sometimes manifest as alternating left and right bundle branch blocks. To the best of our knowledge, there are two previous cases of BVT in the setting of type I myocardial infarction. Our case would be the third and showed a subtle change in the anterior-posterior axis that can be seen in lead V2. The coronary angiography of our patient demonstrated severe multivessel coronary artery disease with complete total occlusion of the proximal dominant right coronary artery, 100% in-stent restenosis of the ostial left circumflex, 40% stenosis of left main, and 90% stenosis of mid left anterior descending artery (LAD). The BVT resolved after two amiodarone boluses followed by a drip. We attempted to transition to oral mexiletine, however, the patient was unable to tolerate the medication due to intractable nausea and vomiting. The patient subsequently underwent high risk coronary artery bypass graft surgery with no further episodes of BVT following revascularization and was discharged after six weeks of hospitalization. Although rare, type I myocardial infarction is an important differential diagnosis of BVT.

## Introduction

Bidirectional ventricular tachycardia is a rare ventricular dysrhythmia with a unique alternating frontal QRS axis and morphology that is commonly associated with digoxin toxicity [1] or catecholaminergic polymorphic ventricular tachycardia (CPVT) [2]. Here, we present an interesting case of BVT in the setting of acute multivessel myocardial infarction.

## Case presentation

The patient is a 59-year-old Caucasian male with a past medical history of coronary artery disease status post percutaneous coronary intervention to left circumflex, hypertension, hyperlipidemia, right carotid artery stenosis status post carotid endarterectomy, heart failure with preserved ejection fraction, diabetes mellitus type 2, diabetic nephropathy with end-stage renal disease on hemodialysis, active tobacco use who was brought in by emergency medical service following presumed cardiac arrest. He first experienced dizziness with hot flashes followed by chest pain while watching television at home. The chest pain was described as a pressure-like sensation that was left-sided in origin with radiation to the left neck and arm. The patient then had a syncopal episode which was witnessed by the patient's wife who called emergency medical service. He was given one dose of 150 mg amiodarone en route for ventricular tachycardia.

**Figure 1:**
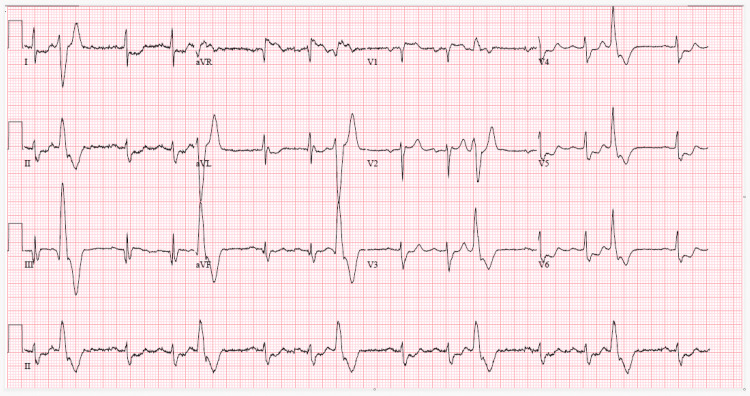
ECG on admission: sinus rhythm with ventricular trigeminy. ECG, electrocardiogram

On arrival at the emergency department, the patient was noted to be in sinus rhythm with ventricular trigeminy at a rate of 89 beats per minute (Figure 1) and blood pressure 142/68 mmHg; other vital signs were within normal limits. 

**Figure 2:**
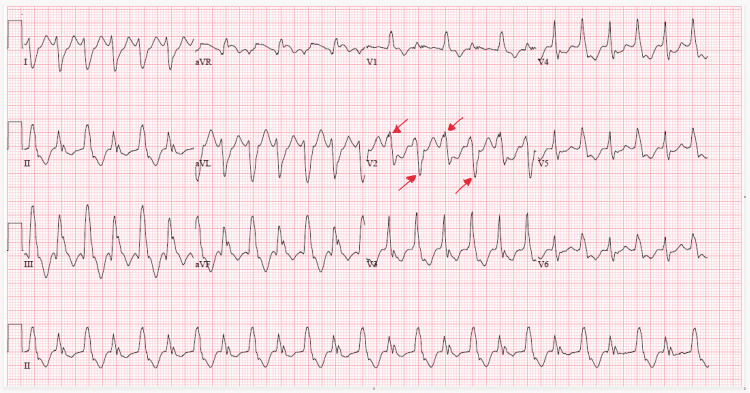
ECG: bidirectional ventricular tachycardia with alternating anterior and posterior axis seen in lead V2. ECG, electrocardiogram

Labs were significant for troponin of 9.68 ng/L (<78) and pro-brain natriuretic peptide of 134,790 pg/mL (0-125). Potassium was 4.6 mEq/L (3.5-5.1) and calcium was 9.3 mg/dL (8.5-10.1). The patient was given amiodarone 150 mg, sodium bicarbonate, calcium gluconate and placed on heparin drip. On the night of the hospitalization, an electrocardiogram (ECG) revealed BVT (Figure 2).

The patient was given two doses of amiodarone 150 mg (in addition to the one dose en route) and BVT subsequently resolved. STAT labs were obtained which showed normal potassium 4.8 mEq/L (3.5-5.1), calcium 8.7 mg/dL (8.5-10.1), and magnesium 2.0 mg/dL (1.8-2.4). 

Prior to the finding of BVT on the night of hospitalization, an echocardiogram was obtained which showed an ejection fraction of 30%-35% with moderate diffuse hypokinesis, grade 2 diastolic dysfunction, mild mitral and tricuspid valve regurgitation. No effusion was demonstrated that could explain the electrical alternans seen on ECG. Urgent coronary angiography revealed 40% stenosis of the left main, 100% in-stent restenosis of the ostial left circumflex, 90% stenosis of mid left anterior descending artery (LAD) and chronic total occlusion (CTO) of the proximal right coronary artery (RCA). The RCA received collaterals from the LAD. Coronary angiography was complicated by monomorphic ventricular tachycardia after crossing the aortic valve that could not be aborted with cardioversion and the patient degenerated into ventricular fibrillation. The patient was eventually resuscitated per advanced cardiac life support (ACLS) protocol and suffered no neurological deficits.

The management of the patient was as follows: he was given boluses of amiodarone and subsequently started on amiodarone drip on arrival at the emergency department due to a finding of ventricular tachycardia. Given decreased ejection fraction and concerns for fluid overload, the patient was taken off amiodarone drip and started on amiodarone by mouth. However, he continued to have recurrent episodes of non-sustained ventricular tachycardia. Mexiletine by mouth (two doses of 150 mg were given, unable to continue due to intractable nausea and vomiting) and IV lidocaine were given as adjunct therapy and BVT rhythm subsequently resolved. The patient underwent three-vessel CABG and had no further episodes of BVT following revascularization.

## Discussion

Bidirectional ventricular tachycardia is a rare and unusual ventricular dysrhythmia that is characterized by a beat-to-beat alternation of the QRS axis. This can sometimes manifest as alternating left and right bundle branch blocks. Our case shows a subtle change in the anterior-posterior axis that can be seen in lead V2. BVT is most commonly seen with digoxin toxicity [1], but can also occur in the setting of CPVT [2], aconite poisoning [3], and myocarditis [4]. 

There are three proposed mechanisms for ventricular tachycardia: re-entry, triggered activity, and automaticity. The mechanism for BVT was initially favored to be secondary to re-entry, which requires two separate conduction circuits in the setting of two electrical scars or two distinct alternating exit sites from one scar [5-8]. Now, it appears that triggered activity through delayed after depolarizations via calcium channels may be a more likely etiology. Studies have shown that mutations and altered function of intracardiac sarcoplasmic reticulum Ca2+ release channels, calsequestrin, and K+ rectifying channels to be the likely culprits [7-8]. These mutations are associated with an increased propensity for delayed afterdepolarizations, which is one of the foundations of the “ping-pong model” of triggered activity. This proposed mechanism described by Baher et al. postulates that there must be exactly two anatomically distinct foci with different thresholds for triggered events [6]. The last proposed mechanism of BVT is abnormal automaticity, wherein alterations in Ca2+ and K+ within cells can increase or decrease the intrinsic pacemaker function [9]. 

To the best of our knowledge, there are two previous cases of BVT in the setting of type I myocardial infarction in the literature. The first case described in 2009 was a patient with severe mid left anterior descending and mid left circumflex disease. BVT was found on admission that terminated spontaneously without any medical intervention [10]. The second case was in 2014 and coronary angiography showed complete occlusion in the right coronary artery disease with mild diffuse disease in the left system. BVT was found during hospitalization that resolved with amiodarone bolus [11].

In the case we present here, coronary angiography showed severe multivessel coronary artery disease with CTO of the proximal dominant right coronary artery, 100% in-stent restenosis of the ostial left circumflex, 40% stenosis of left main, and 90% stenosis of mid LAD. The BVT resolved after amiodarone bolus x2 was followed by a drip. We attempted to convert the drip to Mexiletine by mouth, however, the patient was unable to tolerate the medication due to intractable nausea and vomiting. 

## Conclusions

Bidirectional ventricular tachycardia is a subset of polymorphic ventricular tachycardia and is generally observed in patients with digoxin toxicity or CPVT as mentioned above. We present the third known case of BVT in the setting of type I myocardial infarction. The arrhythmia was successfully treated with amiodarone and the patient subsequently underwent surgical revascularization. Although rare, type I myocardial infarction should be considered to be one of the differentials in the setting of BVT.

## References

[1] Richter S, Brugada P (2009). Bidirectional ventricular tachycardia. J Am Coll Cardiol.

[2] Femenia F, Barbosa-Barros R, Sampaio SV, Arce M, Perez-Riera A, Baranchuk A (2012). Bidirectional ventricular tachycardia: a hallmark of catecholaminergic polymorphic ventricular tachycardia. Indian Pacing Electrophysiol J.

[3] Smith SW, Shah RR, Hunt JL, Herzog CA (2005). Bidirectional ventricular tachycardia resulting from herbal aconite poisoning. Ann Emerg Med.

[4] Berte B, Eyskens B, Meyfroidt G, Willems R (2008). Bidirectional ventricular tachycardia in fulminant myocarditis. Europace.

[5] Ueda-Tatsumoto A, Sakurada H, Nishizaki M (2008). Bidirectional ventricular tachycardia caused by a reentrant mechanism with left bundle branch block configuration on electrocardiography. Circ J.

[6] Baher AA, Uy M, Xie F, Garfinkel A, Qu Z, Weiss JN (2011). Bidirectional ventricular tachycardia: ping pong in the His-Purkinje system. Heart Rhythm.

[7] Levy S, Hilaire J, Clementy J, Bartolin R, Besse P, Gerard R, Bricaud H (1982). Bidirectional tachycardia. Mechanism derived from intracardiac recordings and programmed electrical stimulation. Pacing Clin Electrophysiol.

[8] Lévy S, Aliot E (1989). Bidirectional tachycardia: a new look on the mechanism. Pacing Clin Electrophysiol.

[9] Shenthar J (2015). Unusual incessant ventricular tachycardia: what is the underlying cause and the possible mechanism?. Circ Arrhythm Electrophysiol.

[10] Sonmez O, Gul EE, Duman C, Düzenli MA, Tokaç M, Cooper J (2009). Type II bidirectional ventricular tachycardia in a patient with myocardial infarction. J Electrocardiol.

[11] Wase A, Masood AM, Garikipati NV, Mufti O, Kabir A (2014). Bidirectional ventricular tachycardia with myocardial infarction: a case report with insight on mechanism and treatment. Indian Heart J.

